# Dysregulated balance of Th17 and Th1 cells in systemic lupus erythematosus

**DOI:** 10.1186/ar2964

**Published:** 2010-03-24

**Authors:** Kamini Shah, Won-Woo Lee, Seung-Hyun Lee, Sang Hyun Kim, Seong Wook Kang, Joe Craft, Insoo Kang

**Affiliations:** 1 Department of Internal Medicine, Yale University School of Medicine, S525C TAC, 300 Cedar Street, New Haven, Connecticut 06520, USA; 2 Department of Microbiology, College of Medicine, Seoul National University, 28 Yongon-dong, Chongno-gu, 110-799, Seoul, Republic of Korea; 3 Department of Microbiology, Konkuk University School of Medicine, 322 Danwol-Dong, Chungju, Chungchungbuk-Do 380-701, Republic of Korea; 4 Department of Microbiology, College of Medicine, Kangwon National University, 192-1 Hyoja-Dong, Chunchon, Kangwon-Do 200-701, Republic of Korea; 5 Department of Internal Medicine, College of Medicine, Chungnam National University, 640 Daesa-Dong, Daejeon 301-131, Republic of Korea; 6 Department of Immunobiology, Yale University School of Medicine, 300 Cedar Street, New Haven, Connecticut 06520, USA

## Abstract

**Introduction:**

Interleukin (IL)-17 is a proinflammatory cytokine that is produced largely by a unique CD4^+ ^T-helper (Th) subset called Th17 cells. The development of Th17 cells is suppressed by interferon (IFN)-γ produced by Th1 cells, suggesting cross-regulation between Th17 and Th1 cells. Thus, this study analyzed the balance of CD4^+ ^Th17 and Th1 cell responses in peripheral blood from patients with systemic lupus erythematosus (SLE) and healthy subjects.

**Methods:**

Twenty-five adult patients with SLE and 26 healthy subjects matched for gender and age (± 2 years) were recruited. Peripheral blood mononuclear cells (PBMCs) from patients and healthy subjects were stimulated for 4 h *ex vivo *with phorbol myristate acetate (PMA) and ionomycin. The frequency of CD4^+ ^T cells producing IL-17 and/or IFN-γ was measured by using flow cytometry. Expression of Th17-associated chemokine receptors CCR4 and CCR6 on CD4^+ ^T cells as well as plasma levels of Th17-polarizing cytokines were assessed. Disease activity was evaluated by the SLE disease activity index score (SLEDAI). Unpaired *t *test and Pearson correlation were used for statistical analyses.

**Results:**

Patients with SLE had an increased frequency of CD4^+^IL-17^+ ^T cells compared with healthy subjects. However, the frequency of CD4^+^IFN-γ^+ ^T cells was similar between the two groups, indicating an altered balance of Th17 and Th1 cell responses in SLE. Patients with SLE also had an increased frequency of CD4^+^CCR4^+^CCR6^+ ^T cells that are known to produce IL-17. The frequency of CD4^+^IL-17^+ ^T cells and CD4^+^CCR4^+^CCR6^+ ^T cells correlated with disease activity. In measuring plasma levels of the Th17-polarizing cytokines, levels of IL-6 were higher in patients with SLE than in healthy subjects, although levels of IL-1β, IL-21, IL-23, and transforming growth factor (TGF)-β were not different between the two groups.

**Conclusions:**

We demonstrate an enhanced Th17 cell response that correlates with disease activity in patients with SLE, suggesting a role for IL-17 in the pathogenesis of lupus. Our data indicate that the mechanisms involved in balancing Th1 and Th17 regulation, as well as in producing IL-6, are aberrant in SLE, leading to an increased Th17 response. We suggest that CCR4 and CCR6 expression on CD4^+ ^T cells should be considered as markers of disease activity, and that IL-17 blocking may offer a therapeutic target in SLE.

## Introduction

Systemic lupus erythematosus (SLE or lupus) is an autoimmune-mediated inflammatory disease of unknown etiology [1,2]. The pathologic hallmarks of SLE are altered immune responses to autoantigens with autoantibody production and subsequent tissue injury mediated by the deposition of immune complexes. In lupus, CD4^+ ^T cells are critical drivers of the B-cell-dependent autoantibody response through provision of co-stimulatory signals and cytokines [1,3]. Infiltrates of activated T cells are also found in tissues from affected organs such as the kidneys and skin in lupus [4-6], although their direct role in contribution to tissue injury is unclear.

CD4^+ ^T cells that orchestrate immune responses can be divided into Th1, Th2, and Th17 cells, based on the cytokines they primarily produce (for example, IFN-γ, IL-4, and IL-17, for Th1, Th2, and Th17 cells, respectively) [7]. Differentiation of Th cells is critically dependent on the local cytokine milieu and co-stimulation provided by antigen-presenting cells (APCs) [7]. For instance, TGF-β, IL-1β, IL-6, IL-21, and IL-23 are involved in developing and/or expanding Th17 cells, whereas IFN-γ and IL-4, signature cytokines required for Th1 and Th2 differentiation, suppress Th17 cell development [7,8].

IL-17 is a proinflammatory cytokine that is involved in defending the host against extracellular microorganisms such as fungi [9]. It is produced by several immune-cell subsets including CD4^+^, CD8^+^, and γδ T cells [10-12], as well as by CD3^+^CD4^-^CD8^- ^(double negative or DN) T cells and NK cells [5,13]. IL-17 acts on a broad range of cell types to induce cytokines (IL-6, IL-8, GM-CSF, G-CSF), chemokines (CXCL1, CXCL10), and metalloproteinases [9]. It potently recruits and activates neutrophils by induction of GM-CSF secretion [9], leading to strong inflammatory responses. A role for IL-17 in autoimmunity has been elucidated through mouse studies of experimental autoimmune encephalomyelitis (EAE) and collagen-induced arthritis (CIA), models for multiple sclerosis and rheumatoid arthritis, respectively [14-16], as well as murine lupus models [17,18]. Increased levels of IL-17 also have been found in blood and tissues of patients with inflammatory bowel disease and psoriasis [19,20], suggesting a pathogenic role in human inflammatory diseases. Likewise, patients with SLE have elevated amounts of IL-17 in serum and plasma, with an increased frequency of T cells producing IL-17 in peripheral blood [5,21-24]. Such factors may contribute to the lupus phenotype, because IL-17 acts in conjunction with B-cell activating factor (BAFF) in promoting the survival and proliferation of human B cells and their differentiation into antibody-producing cells [25].

Yet, it remains unknown why IL-17 production is increased in lupus and whether such a finding is related to Th1 cells producing IFN-γ. Determining the balance of Th17- and Th1-cell responses is important, because any enhanced IL-17 activity could be secondary to robust Th-cell responses in general that are typical of SLE, and Th1 cytokine IFN-γ is known to suppress Th17-cell development [7,8]. Here we demonstrate that patients with SLE have an increased frequency of circulating CD4^+ ^T cells producing IL-17, which correlates with disease activity, compared with healthy subjects, whereas both groups maintain similar frequencies of Th1 cells. In addition, plasma levels of IL-6, a cytokine that promotes the development of Th17 cells, are higher in patients with SLE than in healthy subjects. These findings suggest that the balance of Th17 and Th1 responses as well as IL-6 production is dysregulated in SLE, leading to increased IL-17 production from CD4^+ ^T cells, an increase that may contribute to disease pathogenesis.

## Materials and methods

### Patients and healthy individuals

This work was approved by the institutional review committee of Yale University. Twenty-five patients with SLE were recruited from the rheumatology clinic of Yale School of Medicine and Yale New Haven hospital. The diagnosis of SLE was established according to the 1982 revised American College of Rheumatology criteria. Disease activity was evaluated with the SLE disease activity index score (SLEDAI) [26]. Lupus nephritis was diagnosed with renal biopsy. Demographic and clinical characteristics of patients with SLE are summarized in Table 1. Twenty-six healthy individuals matched for gender and age (± 2 years) were recruited as controls. Peripheral blood was collected from human subjects after obtaining informed consent.

**Table 1:** Characteristics of patients with SLE (*n* = 25)

Age, mean ± standard deviation (SD) years	37.7 ± 11.4
Gender, numbers of female/male patients	25/0
Medications	
Number taking methotrexate	5
Number taking azathioprine	5
Number taking mycophenolate mofetil	5
Number taking cyclophosphamide	1
Number taking systemic corticosteroids	14
Prednisone dose, mean ± SD, mg/day	15.89 ± 8.96
Number of patients with nephritis	12

SLE, systemic lupus erythematosus.

### Purification and stimulation of peripheral blood mononuclear cells

Peripheral blood mononuclear cells (PBMCs) were isolated from heparinized peripheral venous blood by using Ficoll-Hypaque gradient (GE Healthcare, Piscataway, NJ). PBMCs were washed with phosphate-buffered saline (PBS) and resuspended in RPMI 1640 media supplemented with 10% fetal calf serum and 1% glutamine/penicillin/streptomycin. Cells were stimulated for 4 hours with PBS (control) or PMA (50 ng/ml; Sigma, St. Louis, MO) and ionomycin (1 μg/ml; Sigma) in the presence of Golgiplug (BD Pharmingen, San Diego, CA) in a tissue-culture incubator at 37°C, as previously done [5,23,27].

### Flow cytometry

PBMCs that had been stimulated with PMA/ionomycin were stained with FITC-conjugated anti-CD3 (eBioscience, San Diego, CA) and PE-Cy5-conjugated anti-CD4 antibodies (BD Pharmingen) followed by fixation and permeabilization by using a Cytofix/Cytoperm kit (BD Bioscience, San Jose, CA) [27]. Cells were then stained with PE-conjugated anti-IL-17 (eBioscience) and APC-conjugated anti-IFN-γ antibodies (BD Pharmingen). Fresh PBMCs were stained with biotin-conjugated anti-CCR6 and PE-conjugated anti-CCR4 antibodies (all from BD Pharmingen) followed by staining with streptavidin-Alexa Fluor 488 [28]. Stained cells were analyzed on a FACSCalibur flow cytometer (BD Biosciences). Collected data were analyzed by using FlowJo software (Tree Star, Ashland, OR).

### ELISA and multiplex cytokine assay

Plasma was separated from heparinized peripheral blood and stored in -80°C for later cytokine assays. Plasma cytokines were analyzed by using commercially available ELISA kits (TGF-β and IL-23 from R&D Systems, Minneapolis, MN; IL-21 from eBioscience) or Bio-Plex Pro human cytokine assay kit (IL-1β, IL-6, IL-10; Bio-Rad, Hercules, CA) in duplicate, according to the manufacturers' instructions. The low limits of detection (pg/ml) for IL-1β, IL-6, IL-10, TGF-β, IL-21, and IL-23 were 0.8, 1.1, 0.9, 31, 31, and 6.8, respectively.

### Statistical analysis

Quantitative data were expressed as the mean ± SD. Unpaired *t *test and Pearson correlation were used for statistical analyses. A value of *P *< 0.05 was considered statistically significant. All statistical analyses were performed by using SPSS statistical software version 16 (SPSS Inc., Chicago, IL).

## Results

### Patients with SLE have an increased frequency of Th17 cells but not Th1 cells in peripheral blood, with an impaired balance of Th17 and Th1 responses

**Figure 1:**
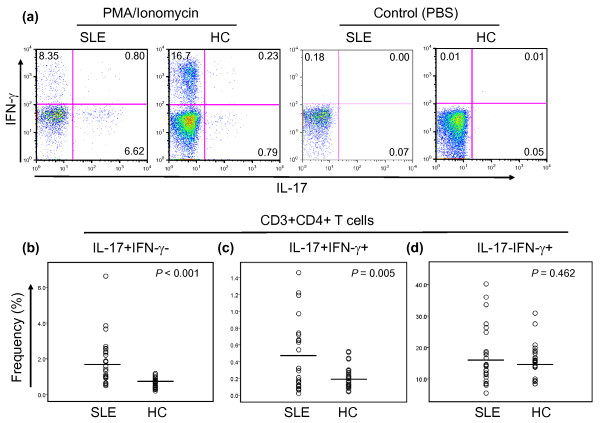
**Patients with SLE have an increased frequency of Th17 cells but not Th1 cells**. PBMCs from patients with SLE (*n* = 25) and healthy control subjects (HC, *n* = 26) were stimulated for 4 hours *ex vivo *with PMA and ionomycin or PBS (control) in the presence of Golgiplug. The frequency of CD4^+ ^T cells producing IL-17 and/or IFN-γ was measured by using flow cytometry. **(a) **Representative dot plots showing CD4^+ ^T cells producing IL-17 and/or IFN-γ. **(b) **The frequency (% of CD4^+^IL-17^+ ^T cells, CD4^+^IL-17^+^IFN-γ^+ ^T cells and CD4^+^IFN-γ^+ ^T cells in patients with SLE and in healthy control subject (HC). Numbers in dot plots indicate the frequency of cells for each quadrant. Bars show the means.

**Figure 2:**
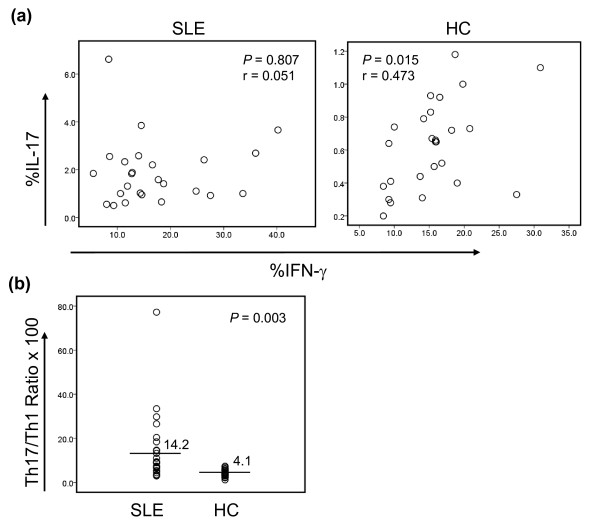
**Correlation of Th17 and Th1 response is dysregulated in patients with SLE**. **(a) **The frequency (% of CD4^+^IL-17^+ ^T cells correlates with the frequency of CD4^+^IFN-γ^+ ^T cells in healthy control subjects (HC, *n* = 26) but not in patients with SLE (*n* = 25). **(b) **Patients with SLE have a higher Th17/Th1 ratio (frequency of CD4^+^IL-17^+ ^T cells/frequency of CD4^+^IL-IFN-γ^+ ^T cells) compared with healthy control subjects. Bars and numbers in (b) indicate the means.

We investigated whether the frequency of Th17 and Th1 cells in patients with SLE differed from that in healthy individuals. After stimulating PBMCs for 4 hours with PMA and ionomycin, we identified CD4^+ ^T cells producing IL-17, IFN-γ, or both, by using flow cytometry (Figure 1a; representative example) [27]. Patients with SLE had an increased frequency of Th17 cells compared with healthy controls (mean ± SD, 1.8 ± 1.26% versus 0.6 ± 0.27%; *P *< 0.001) (Figure 1b), as well as an increased frequency of IL-17 and IFN-γ double-positive cells (0.46 ± 0.41% versus 0.21 ± 0.14%; *P *= 0.005) (Figure 1c), although patients and controls had similar frequencies of Th1 cells(17.1 ± 9.23% versus 15.5 ± 5.47%; *P *= 0.457) (Figure 1d). Of interest, a recent study reported expansion of IL-17-secreting DN T cells in the peripheral blood of lupus patients after long-term (>5 days) *in vitro *stimulation [5]. We also noticed an increased frequency of IL-17-producing cells in CD3^+^CD4^- ^T cells that included DN T cells in patients with SLE compared with healthy controls (1.65 ± 1.45% versus 0.87 ± 0.53%; *P *= 0.016). We next assessed the relation of Th17 to Th1 cells in patients and controls. In the latter, the frequency of IL-17^+ ^cells directly correlated with the frequency of IFN-γ^+ ^cells (*r *= 0.473; *P *= 0.015), although a similar correlation was not observed in patients with SLE (Figure 2a). We next determined the ratio of CD4^+ ^T cells producing IL-17 to the same cells producing IFN-γ because patients with SLE could have an increased frequency of both cell subsets without an alteration in the Th17/Th1 ratio. The ratio of Th17 to Th1 cells was higher in patients with SLE than in healthy controls (Figure 2b). Taken together, these observations indicate that patients with SLE have an aberrant CD4^+ ^T-cell response, resulting in a propensity toward an increased frequency of Th17 cells.

### The frequency of Th17 cells correlates with disease activity in patients with SLE

**Figure 3:**
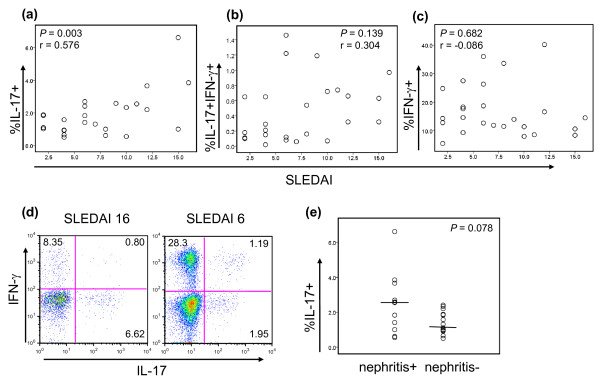
**The frequency of IL-17^+ ^CD4^+ ^T cells correlates with disease activity**. **(a-c) **Relation between SLEDAI score and the frequency (%) of **(a) **CD4^+^IL-17^+ ^T cells, **(b) **CD4^+^IL-17^+^IFN-γ^+ ^T cells and **(d) **CD4^+^IFN-γ^+ ^T cells in patients with SLE (*n* = 25). **(d) **Dot plots showing the frequency of CD4^+ ^T cells producing IL-17 and/or IFN-γ in a patients with SLE at the times of high and low disease activities (SLEDAI score, 16 and 6, respectively). Representative data from two patients with SLE. **(e) **The frequency of CD4^+^IL-17^+ ^T cells in patients with (+, *n* = 11) and without (-, *n* = 14) lupus nephritis. Numbers in dot plots indicate the frequency of cells for each quadrant. Bars show the means.

We determined the relation between the frequency of CD4^+^IL-17^+ ^T cells and disease activity as measured by SLEDAI in patients with SLE. A strong correlation between the two parameters was observed (*r *= 0.597; *P *= 0.003) (Figure 3a). Although the frequency of IL-17 and IFN-γ double-positive cells tended toward a correlation with SLEDAI scores, it was not statistically significant (*r *= 0.304; *P *= 0.138) (Figure 3b). By contrast, no clear correlation was found between the frequency of Th1 cells and disease activity (*r *= -0.086; *P *= 0.682) (Figure 3c). We also determined the correlation of SLEDAI scores with the frequency of CD3^+^CD4^-^IL-17^+ ^T cells which included DN T cells as well as with the frequency of total CD3^+^IL-17^+ ^T cells that contained both CD4^+ ^and DN T-cell subsets. Although a trend was noted toward the positive correlation between the frequency of CD3^+^CD4^-^IL-17^+ ^T cells and SLEDAI scores, it was not statistically significant (*r *= 0.344; *P *= 0.092). The frequency of CD3^+ ^T cells producing IL-17 (total IL-17^+ ^T cells) correlated with SLEDAI scores (*r *= 532; *P *= 0.006). We prospectively analyzed the frequency of CD4^+^IL-17^+ ^T cells in two patients with SLE who had high disease activity at the enrollment. With improved disease activity, the frequency of this cell subset substantially decreased, whereas the frequency of CD4^+^IFN-γ^+ ^T cells increased (Figure 3d).

We next assessed the relation of the Th17-cell response with lupus nephritis and medications. The frequency of these cells tended to be higher in lupus patients with nephritis than in those without nephritis, although the difference was not statistically significant (Figure 3e). No difference was observed in the frequency of CD4^+^IL-17^+ ^T cells between lupus patients who took or did not take glucocorticoids. The frequency of this cell subset also was similar in lupus patients taking and not taking immunosuppressive drugs, including azathioprine, methotrexate, mycophenolate mofetil, and cyclophosphamide (data not shown).

### Patients with SLE have an increased frequency of CD4^+^CCR4^+^CCR6^+ ^T cells in peripheral blood

**Figure 4:**
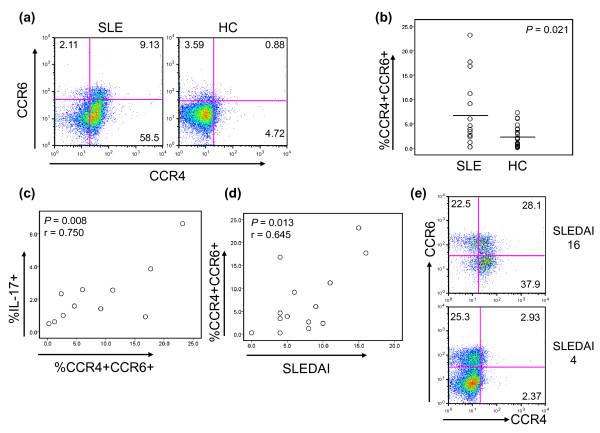
**Patients with SLE have an increased frequency of CD4^+^CCR4^+ ^CCR6^+ ^T cells**. The frequency of CCR4^+ ^CCR6^+ ^CD4^+ ^T cells in peripheral blood was analyzed in patients with SLE and healthy control subjects by using flow cytometry. **(a) **Representative dot plots showing CD4^+ ^T cells expressing CCR4 and CCR6. **(b) **The frequency (%) of CD4^+^CCR4^+^CCR6^+ ^T cells in patients with SLE (*n* = 14) and healthy control subject (HC, *n* = 25). **(c) **Correlation between the frequencies of CD4^+^IL-17^+ ^T cells and CD4^+^CCR4^+^CCR6^+ ^T cells in patients with SLE (*n* = 11). (d) Correlation between the frequency of CD4^+^CCR4^+^CCR6^+ ^T cells and SLEDAI score in patients with SLE (*n* = 14). **(e) **Representative dot plots showing the frequency of CD4^+ ^T cells expressing CCR4 and/or CCR6 in a patients with SLE at the times of high and low disease activities (SLEDAI score, 16 and 6, respectively). Representative data from three patients with SLE. Numbers in dot plots indicate the frequency of cells for each quadrant. Bars show the means.

Previous studies reported that CD4^+ ^T cells producing IL-17 express CCR4 and CCR6 [28]. Thus, we measured the frequency of these cells in the peripheral blood of patients with SLE and in healthy controls (Figure 4a). An increased frequency of CD4^+^CCR4^+^CCR6^+ ^T cells was found in the former group (7.32 ± 7.27% versus 2.18 ± 2.16%; *P *= 0.021) (Figure 4a and 4b), with a correlation with the frequency of Th17 cells (*r *= 0.748; *P *= 0.008) (Figure 4c). Furthermore, a correlation was found between the frequency of CD4^+^CCR4^+^CCR6^+ ^T cells and disease activity (*r *= 0.645; *P *= 0.013) (Figure 4d). In a manner analogous to that of Th17 cells, the frequency of CD4^+^CCR4^+^CCR6^+ ^T cells decreased as disease activity improved (Figure 4e).

### Th17-polarizing cytokines in plasma of patients with SLE and healthy controls

**Figure 5:**
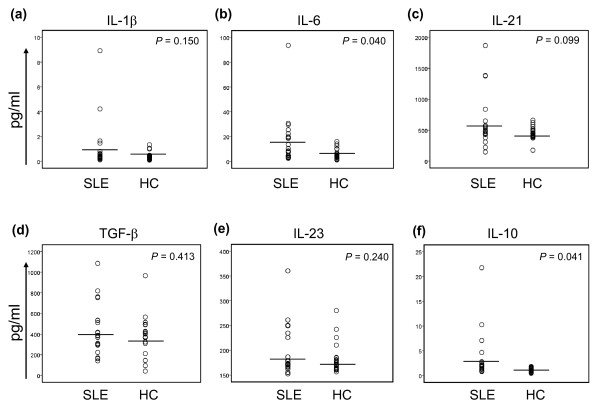
**Plasma levels of Th17-driving cytokines and IL-10 in patients with SLE and healthy controls**. Plasma levels of Th17-polarizing cytokinesg(IL-1β, IL-6, IL-21, TGF-β, and IL-23) and IL-10 were measured in patients with SLE and healthy control subjects (HCs) by using ELISA or multiplex cytokine assay. Bars show the means. Samples from 21 patients with SLE and 24 healthy controls for cytokines except TGF-β (*n* = 20 and *n* = 19 for lupus patients and healthy controls, respectively).

The development of Th17 cells is critically dependent on the cytokine milieu, with IL-1β, TGF-β, IL-6, IL-21, and IL-23 promoting Th17-cell differentiation and expansion [29-34]. Thus, we measured these cytokines in plasma to investigate whether altered production of such cytokine(s) could potentially account for the increased Th17-cell response in SLE. We found increased levels of IL-6 in plasma of lupus patients compared with those in healthy controls (16.03 ± 20.03 pg/ml versus 6.29 ± 4.09 pg/ml; *P *= 0.040) (Figure 5b). Plasma IL-21 levels also tended to be higher in patients than in controls, although the difference was not statistically significant (615.96 ± 425.15 pg/ml versus 450.92 ± 96.67 pg/ml; *P *= 0.099) (Figure 5c); however, plasma levels of other Th17-polarizing cytokines, including IL-1β, IL-23, and TGF-β, were similar between the two groups. As previously reported [35,36], plasma levels of IL-10 were higher in patients with SLE than in healthy controls (3.40 ± 4.79 pg/ml versus 1.11 ± 0.38 pg/ml; *P *= 0.041) (Figure 5f).

## Discussion

We present data demonstrating an enhanced Th17-cell response in patients with SLE compared with healthy controls. More important, the increased frequency of Th17 cells correlated with disease activity, suggesting a potential role for this cytokine in disease pathogenesis. Although the mechanism(s) for these findings remains to be determined, our results indicate that the Th1 and Th17 cell balance, as well as in IL-6 production, are dysregulated in SLE, leading to the increased frequency of CD4^+^IL-17^+ ^T cells in patients.

Although CD4^+ ^T cells are pathogenic in murine, and apparently in human, lupus [2], the contribution of individual Th-cell subsets to disease remains unclear, particularly in humans. Th1 cells appear to promote renal inflammation [37]. Recent studies have reported increased levels of serum or plasma IL-17 as well as an increased frequency of peripheral blood cells producing IL-17 in patients with SLE compared with healthy controls [5,21-24]. IL-17 can be produced from different types of immune cells including CD4^+ ^T cells, CD8^+ ^T cells, and γδ-T cells, as well as DN T cells and NK cells [5,10,11,13]. Of interest, Crispin *et al*. [5] showed expansion of IL-17-secreting DN T cells in the peripheral blood of lupus patients after long-term (>5 days) *in vitro *stimulation. We, conversely, studied CD4^+ ^(not DN) T cells directly *ex vivo*, a much better reflection of the *in vivo *situation than long-term culture, a situation that can artificially expand a potentially trivial population, or conversely, lead to contraction of an expanded population. We also noticed an increased frequency of IL-17-producing cells in CD3^+^CD4^- ^T cells that included DN T cells in patients with SLE compared with healthy controls. Yang *et al*. [23] revealed an increased frequency of CD3^+^CD8^-^IL-17^+ ^T cells in the blood of lupus patients compared with healthy controls. The expanded population of CD3^+^CD8^-^IL-17^+ ^T cells identified by this study must have included DN T cells. Of interest, in the same study, lupus patients had increased *IFN-γ *gene expression in PBMCs and higher serum levels of the same cytokine compared with healthy controls, as measured by quantitative PCR and ELISA, respectively. IFN-γ has multiple cellular sources including CD4^+^, CD8^+ ^T cells and innate immune cells, including macrophages and NK cells. Thus, these assays could not tell whether such findings were secondary to increased IFN-γ production from CD4^+ ^T cells. This is a critical point, because any increased frequency of IL-17-producing T cells could be secondary to enhanced Th function in general. Furthermore, numbers of Th17 cells should be investigated with an analysis of Th1 cells, given that IFN-γ can suppress the development of IL-17-producing cells [8]. Indeed, our study demonstrated a dysregulated balance between Th1 and Th17 cells in SLE, a novel finding. Because very few CD8^+ ^T cells produced IL-17 in PBMCs from patients with SLE and healthy controls after 4 hours of PMA and ionomycin stimulation (data not shown), our work indicates that increased IL-17 production in patients with SLE is contributed predominantly by CD4^+ ^T cells and DN T cells.

We found a strong positive correlation between the frequency of Th17 cells and disease activity. Although this finding suggests that the increased IL-17 production in lupus is biologically relevant, the precise role for this cytokine in the pathogenesis of lupus has yet to be elucidated. A recent study reported that IL-17 alone or in combination with BAFF promoted the survival and proliferation of human B cells and their differentiation into antibody-producing cells [25]. This observation provides a novel insight into understanding the pathogenic role for IL-17 in lupus because aberrant B-cell immunity with autoantibody production is essential for tissue damage and inflammation in human and murine lupus. Of interest, we found increased levels of plasma IL-10, as previously reported [35]. The synthesis of IL-17 may be linked to increased B-cell production of IL-10 in lupus that also potently promotes humoral immunity [2]. In our study, lupus patients with nephritis had a trend toward an increased frequency of CD4^+^IL-17^+ ^T cells and CD3^+^CD4^-^IL-17^+ ^T cells compared with those without nephritis. Infiltrates of IL-17 producing T cells, including CD4^+ ^and DN T cells, have been found in lupus nephritis. In addition, *IL17 *gene expression was detected in T cells infiltrating the kidneys and in urine sediments of lupus patients [38,39]. These findings support the possible pathologic significance of our findings [5].

The mechanism for increased IL-17 production in patients with SLE is unclear. Although this finding could be secondary to increased CD4^+ ^T-cell responses in general, the results of our study showed that the frequency of Th17 but not Th1 cells was increased in patients with SLE compared with healthy controls. Furthermore, the positive correlation between the frequencies of Th17 and Th1 cells that was found in healthy controls was disrupted in lupus patients. These observations indicate that the balance of Th17 and Th1 cell responses is dysregulated in SLE, leading to enhanced Th17 cell response. Thus, we explored a potential role for polarizing cytokines in promoting IL-17 production in SLE, because the development of Th subsets is critically dependent on the cytokine milieu. Plasma levels of IL-6 were higher in patients with SLE than in healthy subjects, suggesting the possible involvement of this cytokine in enhancing the Th17-cell response we observed. In line with this finding, increased circulating levels of IL-6 are found in patients with SLE [35]. We also noticed that patients with SLE had a trend for increased plasma levels of IL-21, a cytokine that can be produced from Th17 cells and promotes both humoral and Th17 immune responses [29,40]. In contrast to IL-6, plasma levels of IL-1β, IL-23, and TGF-β were similar between the two groups. We believe that further studies are warranted to determine the mechanism for increased IL-17 production from CD4^+ ^T cells in human lupus.

Several cell-surface molecules were reported as potential markers for Th17 cells. To date, the best-known molecules are CCR4 and CCR6 [28]. We noticed a strong correlation between the frequencies of CD4^+^IL-17^+ ^T cells and CD4^+^CCR4^+^CCR6^+ ^T cells in the peripheral blood of lupus patients. The frequencies of both cell subsets correlated with disease activity, as measured cross sectionally and prospectively, raising the possibility of using such cell measurements in assessing disease activity in patients with SLE. Clinical studies with large numbers of patients will help address this point. In contrast to our observation, a recent study did not find an increased frequency of CCR4^+^CCR6^+ ^T cells in peripheral blood of lupus patients [5]. Although the reason for this discrepancy is not clear, it could be related to the fact that this study noticed an increased frequency of DN T cells but not CD4^+ ^T cells producing IL-17. Of interest, CD4^+^CCR4^+^CCR6^- ^T cells also appeared to expand in active lupus patients (Figure 4a and 4e). However, CD4^+^CCR4^+^CCR6^+ ^T cells and CD4^+^CCR4^+^CCR6^- ^T cells have different capacities for cytokine production. The former subset, but not the latter, can produce large amounts of IL-17 [28].

## Conclusions

In summary, our study provides evidence of a role for IL-17 in the pathogenesis of SLE, with the demonstration of an increased frequency of Th17 cells in the peripheral blood of lupus patients, and a correlation of the frequency of these cells with disease activity. Although the mechanism underlying our findings is yet to be determined, it appears that factor(s) involved in balancing Th17 and Th1 cell responses as well in producing IL-6 are dysregulated in SLE. Our data offer a scientific rationale for exploring the utility of Th17 cells, as well as Th17-associated molecules CCR4 and CCR6 as biologic markers for disease activity in human lupus. Our observations also raise the possibility of anti-IL-17 therapy in controlling disease activity in SLE.

## Abbreviations

APCs: antigen-presenting cells; BAFF: B-cell activating factor; CIA: collagen-induced arthritis; DN: double negative; EAE: experimental autoimmune encephalomyelitis; PBMCs: peripheral blood mononuclear cells; PBS: phosphate-buffered saline; PMA: phorbol myristate acetate; SLE or lupus: systemic lupus erythematosus; SLEDAI: SLE disease activity index score; Th: T helper.

## Competing interests

The authors declare that they have no competing interests.

## Authors' contributions

IK and JC had full access to all of the data in the study and took responsibility for the integrity of the data as well as for manuscript preparation. KS performed most of the experiments, data analysis, and manuscript preparation. WWL, SWK, SHK, and SHL participated in study design, data acquisition, and analysis. All authors read and approved the final manuscript.
